# Fabrication and Characterization of Benzhydroxamic Acid-Loaded Dissolving Microneedles Using a 3D-Printing-Assisted Mold Fabrication Approach

**DOI:** 10.3390/mi17091006

**Published:** 2026-08-26

**Authors:** Arjun Gokulan Manivannan, Narayanan Jayasankar, Bhupendra G. Prajapati, Karan Prajapati, Suhaskumar Patel

**Affiliations:** 1 Department of Pharmacology, SRM College of Pharmacy, Faculty of Medicine and Health Sciences, SRM Institute of Science and Technology, Kattankulathur, Chengalpattu 603203, Tamil Nadu, India; 2 Department of Pharmaceutics, Parul Institute of Pharmacy, Faculty of Pharmacy, Parul University, Waghodia, Vadodara 391760, Gujarat, India; 3 Department of Industrial Pharmacy, Faculty of Pharmacy, Silpakorn University, Nakhon Pathom 73000, Thailand; 4 Research and Development, Amneal Pharmaceutical, Piscataway, NJ 08854, USA; kp7867@zohomail.com; 5 Formulation Research and Development, Amneal Pharmaceutical, Piscataway, NJ 08854, USA

**Keywords:** benzhydroxamic acid, dissolving microneedles, 3D printing, transdermal drug delivery, PVA, PVP, drug permeation

## Abstract

Dissolving microneedles offers a minimally invasive approach for transdermal drug delivery by facilitating drug transport across the stratum corneum while overcoming several limitations associated with conventional routes of administration. Benzhydroxamic acid has demonstrated biochemical and computational evidence associated with inflammatory and pain-related pathways; however, its incorporation into a dissolving microneedle platform has not been extensively explored.This study aimed to fabricate and characterize benzhydroxamic acid-loaded dissolving microneedles using a 3D-printing-assisted mold fabrication approach for transdermal drug delivery. A stereolithography-based 3D-printed master mold was used to prepare a reverse polydimethylsiloxane mold. Benzhydroxamic acid-loaded dissolving microneedles were fabricated using a PVA/PVP polymeric matrix and evaluated for their physicochemical, mechanical, insertional, and drug-delivery characteristics. The developed microneedles exhibited shear-thinning behavior, uniform morphology, and satisfactory mechanical properties, with a compression force of 3.5 ± 0.01 N/needle and tensile strength of 3.84 ± 0.21 MPa. The formulation demonstrated a drug-loading efficiency of 94.6 ± 0.35% and effective insertion into the Parafilm^®^ M skin-simulant model. In vitro drug release reached 97.24% over 24 h, while ex vivo skin permeation reached 94.83% over 24 h. FTIR and XRD analyses indicated successful incorporation of benzhydroxamic acid into the PVA/PVP matrix without major evidence of drug–polymer incompatibility. The findings demonstrate the feasibility of incorporating benzhydroxamic acid into a PVA/PVP dissolving microneedle platform using a 3D-printing-assisted mold fabrication approach. The developed system exhibited suitable physicochemical and mechanical characteristics, efficient drug loading, effective insertion, and satisfactory in vitro and ex vivo drug-delivery performance, supporting its potential as a transdermal drug delivery platform.

## 1. Introduction

The transdermal drug delivery system has been evolving over a period of years. They are boosted to produce controlled drug release, reduce dose frequency, improve patient compliance, and escape hepatic first-pass metabolism. These benefits levelled them above the conventional oral and parenteral routes of delivery [1]. The conventional route of oral administration lacks certain approaches, such as fluctuations in the plasma concentration and gastrointestinal irritation, and during long-term therapy, they produce varied adverse effects [2]. These failures in conventional techniques necessitated the requirement of better upgraded futuristic therapy to produce efficient therapeutic results. Though transdermal drug delivery overcomes most of these difficulties, it must surpass the highly organized structure of the stratum c. This layer is the outermost part of the skin, which protects the skin from the penetration of foreign particles into the skin; therefore, to produce an efficient therapeutic action, the drug must pass through the stratum corneum [3]. Hereby designing an effective and reproducible transdermal delivery system remains the biggest challenge during formulation development.

One of the techniques growing in recent years involves a microneedle-based drug delivery approach, which facilitates transient disruption of the stratum corneum, making a microscale pathway to enhance the penetration of drugs reaching the deeper receptors and blood vessels [4]. There are various types of microneedles being developed over a period, but interestingly, dissolving microneedles grab attention due to their unique feature of dissolving completely within the skin upon their administration and disregarding concerns related to needle breakage or sharp biohazardous waste. They also provide additional benefits such as minimal invasive administration, enhanced patient acceptability, and support for localized as well as sustained drug release behavior [5]. These advantages prove dissolving microneedles suitable for controlled transdermal delivery applications.

Three-dimensional (3D) printing-assisted micromolding has emerged as an enabling strategy for the rapid prototyping of microneedle master molds, offering control over needle geometry, array configuration, and fabrication reproducibility [6,7,8,9]. In this approach, digitally designed master structures can be fabricated using high-resolution printing and subsequently replicated into polymeric molds for the preparation of dissolving microneedles. Although 3D-printing-assisted micromolding is an established fabrication strategy, it provides a practical approach for producing customized microneedle geometries and investigating new drug–polymer combinations.

Polymer selection plays an important role in determining the mechanical strength, dissolution behavior, and drug-release characteristics of dissolving microneedles. Hydrophilic polymers such as poly(vinyl alcohol) (PVA) and polyvinylpyrrolidone (PVP) are widely used because of their film-forming ability, mechanical properties, and water-soluble characteristics. The combination of PVA and PVP can provide a balance between mechanical integrity and dissolution behavior, making this polymeric combination suitable for dissolving microneedle fabrication [6,7].

Benzhydroxamic acid (BHA) is a hydroxamic acid derivative containing a metal-binding hydroxamate functional group. Hydroxamic acids are recognized as important metal-binding pharmacophores and can interact with the catalytic sites of zinc-dependent enzymes, including histone deacetylases and matrix metalloproteinases [10,11,12]. Benzhydroxamic acid-based structures have therefore been investigated in medicinal chemistry, particularly as zinc-binding groups in histone deacetylase inhibitors [13]. Experimental structural studies have also demonstrated direct interaction of BHA with biologically relevant proteins. For example, the crystal structure of BHA in a complex with horseradish peroxidase demonstrated a defined binding site and hydrogen-bonding interactions between BHA and residues within the enzyme active site [11,13,14]. In addition, BHA has been investigated for its interaction with prostaglandin-endoperoxide synthase 2 (PTGS2; also known as prostaglandin H_2_ synthase-2, PGHS-2), providing biochemical evidence for its interaction with an enzyme associated with inflammatory signaling [13]. These findings establish that BHA possesses measurable biochemical and protein-interaction properties, providing a basis for its further investigation as a candidate small molecule for drug-delivery development [10,13,14].

More specifically, our previous computational investigation explored BHA as a potential multi-target lead for neuropathic pain. Network pharmacology analysis identified molecular targets and pathways associated with inflammatory and pain-related signaling, while molecular docking and molecular dynamics analyses supported favorable interactions of BHA with prostaglandin-endoperoxide synthase 2 (PTGS2) and matrix metalloproteinase-9 (MMP-9) [13]. These findings provided a hypothesis-generating molecular rationale for further investigation of BHA in the context of neuropathic-pain-related drug delivery. These findings provide a hypothesis-generating molecular rationale for further investigation but do not establish the therapeutic efficacy of BHA in neuropathic pain.

To the best of our literature search, benzhydroxamic acid (BHA) has not previously been investigated as the active pharmaceutical ingredient in a dissolving microneedle platform. Published studies involving BHA have primarily focused on its biochemical interactions, protein binding, and computationally predicted pharmacological relevance, including interactions with inflammation-associated targets such as PTGS2 and MMP-9 [13]. However, these studies have not addressed the development of a transdermal BHA delivery system or its incorporation into a dissolving microneedle matrix. The present study, therefore, applies an established 3D-printing-assisted micromolding approach to develop a BHA-loaded PVA/PVP dissolving microneedle system, with the novelty residing in the incorporation and delivery of BHA rather than in the fabrication technology itself. The developed system was evaluated for rheological behavior, morphology, dimensional uniformity, mechanical properties, insertion capability, drug-loading efficiency, physicochemical compatibility, and in vitro and ex vivo drug-delivery performance.

## 2. Material and Methods

### 2.1. Materials

Benzhydroxamic acid (≥99.0%), polyvinylpyrrolidone (PVP), polyvinyl alcohol (PVA), glycerol (99.5%), Parafilm^®^ M (~130 µm thickness), and phosphate-buffered saline (PBS) (pH 7.4) were procured from Sigma-Aldrich Chemicals Private Limited, Bangalore, India. All the other reagents used were of analytical grade.

### 2.2. Methods

#### 2.2.1. Design and Fabrication of 3D-Printed Master Microneedle Mold

**Figure 1:**
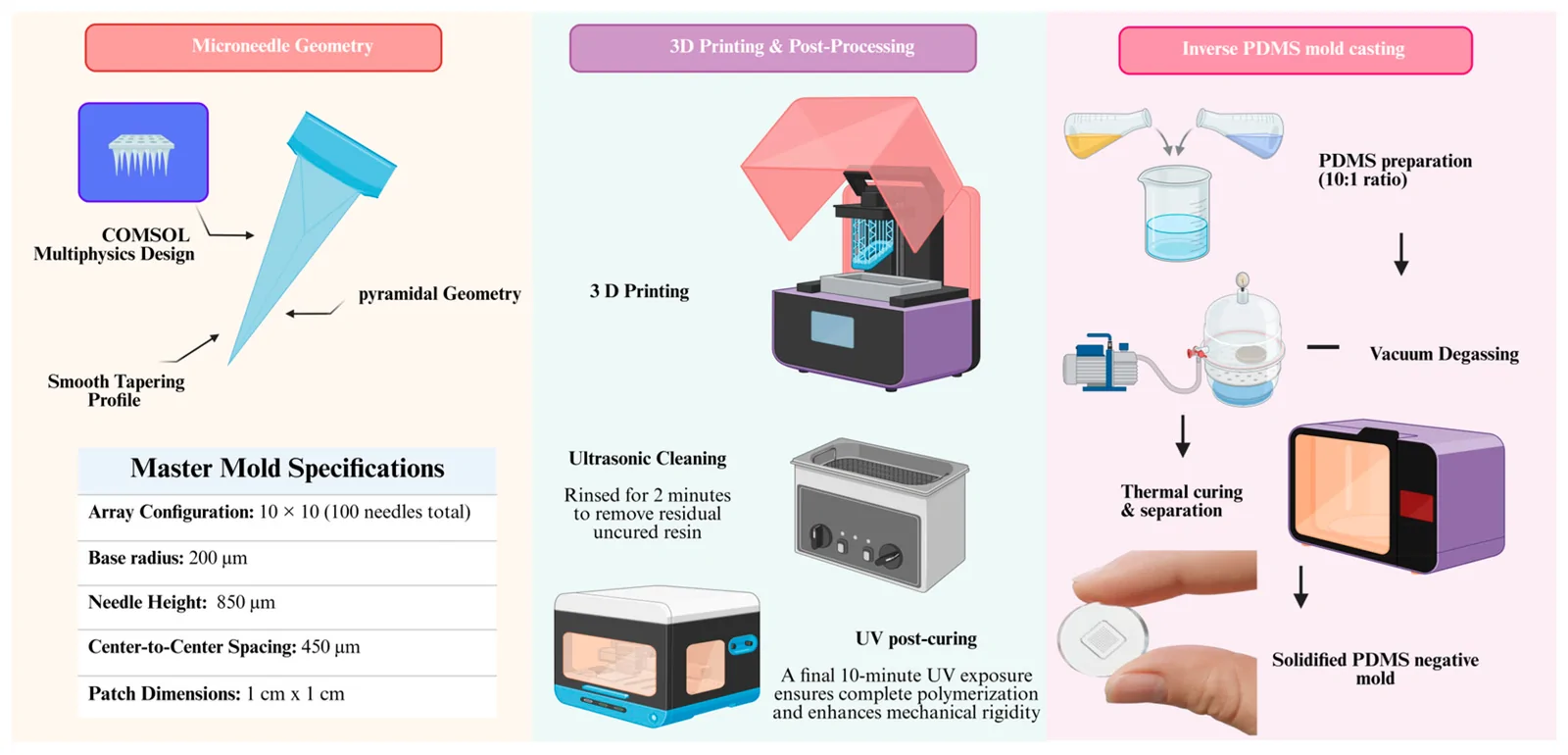
Schematic representation of the 3D-printed master microneedle mold and its replication into a negative polydimethylsiloxane (PDMS) micromold, showing the designed microneedle geometry and array configuration.

A three-dimensional (3D)-printed master microneedle mold was fabricated using stereolithography (SLA) based on a computer-aided design (CAD) model. The microneedle geometry was designed using COMSOL Multiphysics software version 6.3 to define the desired needle architecture and dimensions. The microneedle geometry consisted of a conical structure with a needle length of 850 µm, a base radius of 200 µm, and an apex radius of 15 µm. The array contained 100 microneedles arranged in a 10 × 10 configuration, with an inter-needle spacing of 450 µm. The selected geometry was designed considering skin insertion, mechanical stability, and reliable micromold replication. The needle height of 850 µm and apex radius of 15 µm were selected to provide sufficient needle length and tip sharpness for insertion, while the base radius of 200 µm was selected to provide adequate structural support. An inter-needle spacing of 450 µm was used to provide sufficient separation between adjacent needles and facilitate uniform cavity replication. Previous studies have demonstrated that microneedle height, tip dimensions, base dimensions, and inter-needle spacing influence insertion capability, mechanical strength, penetration depth, and array integrity [15]. Therefore, the selected geometry was designed to balance sufficient tip sharpness and penetration capability with adequate mechanical stability and reproducible micromold replication. The CAD model was exported in stereolithography (STL) format and processed using PreForm software version 3.56 (Formlabs, Somerville, MA, USA) before fabrication using a Formlabs SLA 3D printer. Following printing, the master mold was immersed in isopropyl alcohol and subjected to ultrasonication for 10 min to remove residual uncured photopolymer resin. The cleaned master mold was subsequently subjected to ultraviolet post-curing using a Form Cure system to improve the dimensional stability and mechanical rigidity of the printed structure. The resulting positive master mold was then used as the template for the fabrication of the negative PDMS micromold [16,17]. The process of the fabrication is illustrated in Figure 1.

#### 2.2.2. Development of Reverse PDMS Mold and Benzhydroxamic Acid-Loaded Bilayer Dissolving Microneedles

The negative PDMS micromold was prepared by replica molding using the 3D-printed master mold as the template. PDMS elastomer and curing agent were mixed at a 10:1 ratio to obtain a homogeneous prepolymer mixture. The mixture was subjected to vacuum degassing to remove entrapped air bubbles and was then carefully poured over the 3D-printed master mold to completely cover the microneedle structures. The PDMS-coated master mold was placed in a hot-air oven at 60 °C for 4 h to allow curing of the PDMS. After complete curing, the PDMS mold was carefully separated from the master mold, generating a negative mold containing microneedle-shaped cavities. The resulting PDMS mold was subsequently used for casting the BHA-loaded dissolving microneedle formulation.

**Figure 2:**
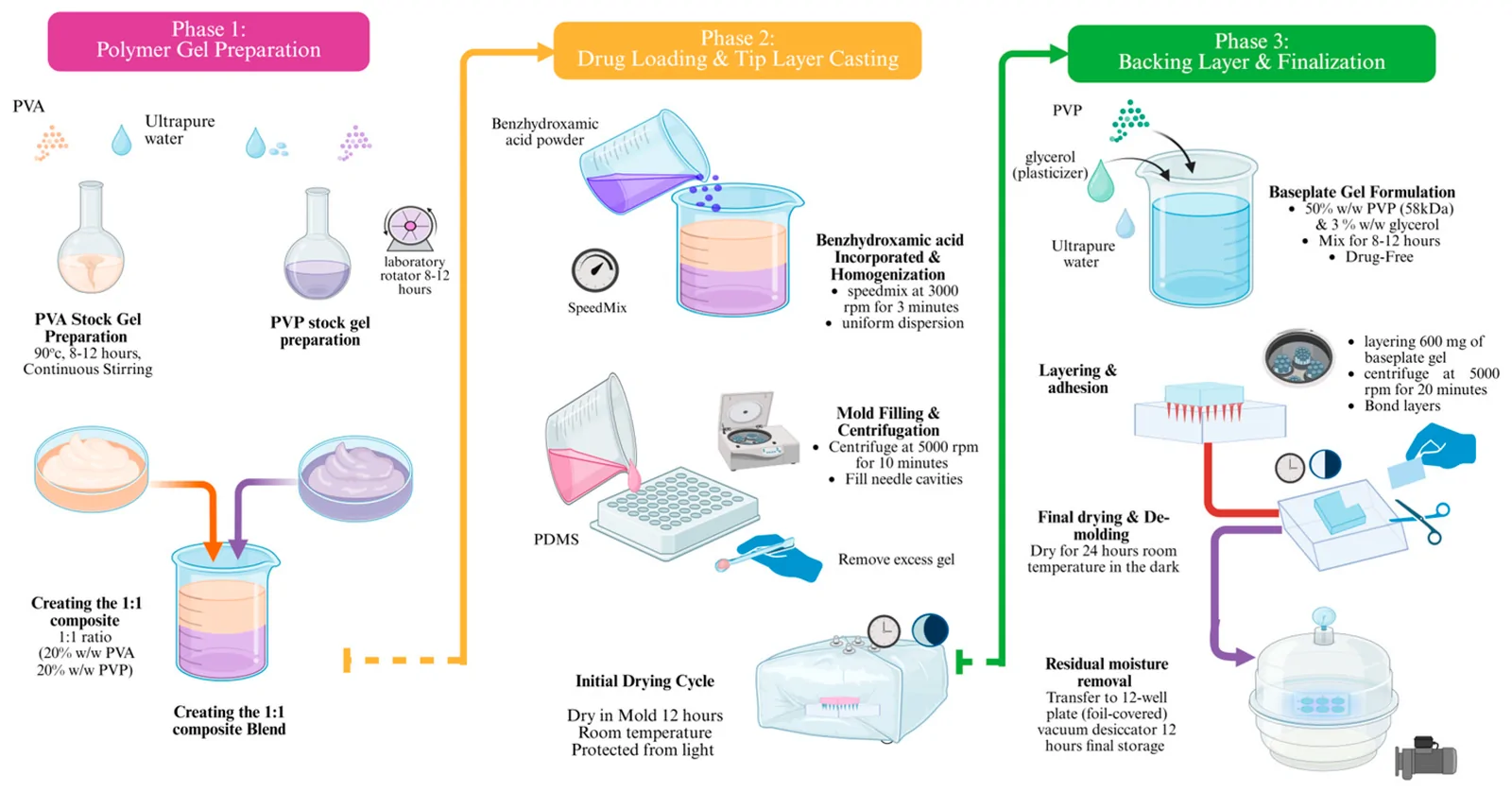
Schematic representation of the fabrication workflow for BHA-loaded bilayer dissolving microneedles using the negative PDMS micromold.

The BHA-loaded dissolving microneedles were prepared by a two-step micromolding process consisting of microneedle-tip formation followed by backing-layer formation. For preparation of the microneedle-tip formulation, PVA (20% *w*/*w*) and PVP (20% *w*/*w*) were dissolved in distilled water and mixed using a magnetic stirrer to obtain a homogeneous polymeric solution. The PVA/PVP composition and the corresponding processing conditions were adopted from our previously developed pregabalin-loaded dissolving microneedle formulation, in which this polymeric composition was established as suitable for microneedle fabrication and processing [15]. BHA was subsequently incorporated into 100 mg of the PVA/PVP polymeric formulation containing 100 mg of distilled water and mixed thoroughly to achieve uniform drug distribution. The drug-loaded polymeric formulation was then dispensed onto the PDMS mold and subjected to low-speed centrifugation to facilitate filling of the microneedle cavities and removal of entrapped air. Excess formulation present on the surface of the mold was carefully removed, and the filled mold was allowed to dry at ambient temperature for 24 h. After partial drying of the microneedle layer, a drug-free backing layer containing PVP (50% *w*/*w*) and glycerol (3% *w*/*w*) was applied over the microneedle array and allowed to stabilize for a further 24 h. The processing parameters were retained from the previously developed pregabalin-dissolving microneedle formulation [15], as these conditions provided suitable polymer solution handling, mold filling, and formation of structurally intact dissolving microneedles [16,17]. Following complete drying, the bilayer BHA-loaded dissolving microneedle patch was carefully demolded and stored in a desiccator until further characterization. The fabrication process of BHA-loaded bilayer dissolving microneedles using the negative PDMS micromold is shown in Figure 2.

### 2.3. Characterization of Dissolving Microneedle

#### 2.3.1. Rheology Analysis

The Brookfield Rheometer (R/S-CPS, P25 DIN plate, Brookfield Engineering Laboratories, Middleboro, MA, USA) was employed to investigate the rheological behavior of the PVP solution, PVA solution, and benzhydroxamic acid-loaded PVA/PVP solution. Parallel plates with a 25 mm diameter and a 1 mm plate spacing, having a shear range of 0.1–100 s^−1^, were employed. The rheological behavior was assessed using approximately 0.6 mL of each sample [18]. The measurement was performed using one representative sample (*n* = 1) under ambient laboratory conditions.

#### 2.3.2. Morphology

Microneedle structures were examined using a Field Emission Scanning Electron Microscope (FESEM) (JEOL, IT800HL). Carbon-conductive adhesive tapes were used to firmly place the sectioned MN on an aluminum vertical stub. To enhance the surface conductivity, the MNs were coated using gold sputtering for about a minute [19]. Further images were photographed at a low voltage of 15 kV. FESEM micrographs were depicted at 30× magnification.

#### 2.3.3. Dimensional Uniformity

The uniformity of the MN patches was calibrated through Vernier caliper [20]. The mean thickness ± standard deviation was computed using 3 selected independent patches. The experiment was performed in triplicate under ambient laboratory conditions. Each patch was weighed separately on an electronic digital balance to determine weight variation among the patches, and their mean value ± standard deviation was observed.

#### 2.3.4. Folding Endurance

The folding endurance is the ability of the object to withstand folding and rupture. The patch was folded back and forth until it was ruptured. The total count of folds necessitated to rupture the patch was noted; further, their mean value and standard deviation were determined [21]. This assessment was utilized to determine the flexural capacity of the patch on administration and storage. The test was performed using three independent microneedle patches (*n* = 3) under ambient laboratory conditions.

#### 2.3.5. Swelling Study

The hydration kinetics of the polymeric MN matrix were assessed using the swelling behavior analysis. The MN patches were oven-dried to obtain 3 consecutive weights, which were taken as the basal weight (W_0_). The patches were solubilized in 10 mL of PBS at a pH of 5.5 and retained at 32 ± 0.5 °C. The patches were withdrawn from PBS at predetermined intervals, gently blotted to remove any wetness on the surface of the patch, and weighed, denoting (Wt) [22]. The swelling index was evaluated using the formula

% swelling index = Wt − W_0_/W_0_ × 100

The experiment was performed using three independent microneedle patches (*n* = 3) at 32 ± 0.5 °C.

#### 2.3.6. Mechanical Strength

Compression studies were carried out using an applied axial load (digital force gauge (ZTA-50N)) in compression mode. Double-sided adhesive tape was employed to secure the MNs onto the platform, ensuring that the needle points were aligned with the probe axis. The flat stainless-steel probe was moved downward at a constant rate of 1 mm/s with a low trigger force (~0.05 N) until a force of 32 N was achieved. This force measures the maximum average force that a person can exert when compressing an MN against the skin. The sudden decline in the force indicates the rupture of the needle. Dividing the maximum force exerted by the number of needles ruptured provides the force exerted on each needle. The initial height of the needles was compared to the heights of the needles upon compression, applying optical microscopy to determine the percentage of deformation in the needle height [23]. The experiment was performed using three independent microneedle patches (*n* = 3) under ambient laboratory conditions.

#### 2.3.7. Tensile Strength

The tensile strength signifies the material’s ability to resist breaking when it is subjected to a tensile (pulling) force. A TA.XT.PLUS Texture Analyzer with an A/TG Tensile Grip sensor was employed to measure the tensile stability of the patch [24]. The patch was held between the instrument’s upper and lower jaws. The upper clamp was gradually advanced at a constant rate of 0.50 mm/s after the patch was positioned correctly. This movement lasted until the patch eventually fragmented. The material’s extensibility (its capacity to extend before breaking) and tensile strength were recorded during the process. The experiment was performed using three independent microneedle patches (*n* = 3) under ambient laboratory conditions, and the results were expressed as mean ± SD.

#### 2.3.8. In Vitro Insertion Study

To evaluate the insertional studies, a marketed polymeric film (Parafilm^®^ M film) was utilized as a skin stimulant. To remodel the skin morphology, the Parafilm^®^ M (~750 µm) was stacked in 6 layers [25]. Thereafter, the microneedles were inserted at a force of 40 N for 30 s using an insertion applicator under ambient laboratory conditions. The microneedle was removed, and the number of holes developed was quantified using microscopic analysis, and percentage insertion efficacy was derived.

Percent Insertion = Number of holes created in the Parafilm × 100/Total No. of Microneedles

#### 2.3.9. Analytical Method for Benzhydroxamic Acid Quantification

The benzhydroxamic acid was quantified using UV-visible spectroscopy (Shimadzu, Kyoto, Japan). To assess the maximum absorption wavelength (λmax), the drug solution was scanned at a range of 200–400 nm. The maximum absorbance was displayed at 230 nm. The benzhydroxamic acid drug solution was prepared using phosphate-buffered saline (PBS) at pH 7.4, which was then diluted again using PBS to acquire the working standard solutions. The calibration curve was constructed at a concentration range of 2–12 ug/mL. At 230 nm, the absorbance of all the standard solutions was recorded, consisting of PBS as the blank [26]. The calibration curve was generated by plotting absorbance against the concentration, followed by determining the regression equation and correlation coefficient. The calibration curve was examined to quantify the benzhydroxamic acid during drug loading potency and in vitro drug release studies.

#### 2.3.10. Drug Loading Efficiency

The drug loading efficiency was analyzed using a UV-visible spectrophotometer. Firstly, 1 mL of phosphate-buffered saline (PBS, pH 7.4) was employed to solubilize the MN patch via sonication for 30 min to establish complete extraction of benzhydroxamic acid from the polymer matrix. Further, it was purified using a 0.2 μm filter to elucidate undissolved particles. The concentration of benzhydroxamic acid was determined in the resultant solution [27]. The test was conducted in triplicate to minimize the error. The calibration curve mentioned in 2.3.9 was used to determine the drug content present in the MN. Percentage drug loading potency was derived using a formula, and the theoretical drug concentration was taken as the initial quantity of benzhydroxamic acid incorporated during formulation. The analysis was performed using three independent microneedle patches (*n* = 3), and the results were expressed as mean ± SD.

% Drug loading efficiency = The concentration of benzhydroxamic acid in the microneedle patch/The theoretical concentration of benzhydroxamic acid in the dry polymer × 100

#### 2.3.11. FTIR

Fourier transform infrared spectroscopy (FT-IR) (SHIMADZU IRTracer-100 (range 400–4000 cm^−1^)) analysis was performed to evaluate the molecular configuration of the drug and MN formulation. The dried MN patch powder via mortar and pestle, pure drug, and polymers were scanned at a resolution of 4 cm^−1^ [28]. The spectrum of the MN formulation was compared across the spectrum and to the major peaks of the functional groups obtained from the pure drug and polymers. The compatibility of the MN formulation and polymers was examined through FTIR analysis.

#### 2.3.12. X-Ray Diffraction (XRD)

The XRD was analyzed using an X-Ray Diffractometer (XRD) (powder and thin films) (PANalytical, Almelo, The Netherlands). The samples were exposed to Cu Kα radiation (λ = 1.54178 Å, 40 kV, and 40 mA). They were then carefully placed in a uniform pattern to ensure precise diffraction. The diffraction patterns were measured at refined temperatures over a 2θ range of 2° to 60° in continuous mode. The step size analysis was carried out at 0.05°/s to secure high resolution and optimize the peaks obtained in the time between the steps [29]. The diffraction patterns detect the alteration in the physical characteristics between the polymer, benzhydroxamic acid, and MN matrix.

#### 2.3.13. In Vitro Release

An in vitro drug release study was conducted to measure the concentration of drug released at established intervals of time in an optimized condition. Prior to in vitro release analysis, the drug loading potency is recorded to explore the concentration of drug loaded in the MN arrays. The in vitro release study was evaluated by dissolving the patches in 50 mL PBS consisting of 3% *w*/*v* Tween 20. The entire system was maintained at 37 °C, and at preset time intervals, 1 mL of solution was withdrawn, and the concentration of drug present in it was measured using a UV-visible spectrophotometer. Hence, the rate of drug release at various time intervals was determined [29,30]. The drug-release data were further analyzed using zero-order, first-order, Higuchi, and Korsmeyer–Peppas kinetic models. The coefficient of determination (R^2^) was used to compare the goodness of fit of the different kinetic models. For the Korsmeyer–Peppas model, the release exponent (*n*) was calculated from the slope of the linear regression of log(Mt/M∞) versus log(time) using the initial portion of the release profile. The zero-order model was expressed as Q_t_ = Q_0_ + K_0_t, the first-order model as log (Q∞ − Q_t_) = log Q∞ − K_1_t/2.303, and the Higuchi model as Q_t_ = K_H√t. The Korsmeyer–Peppas model was expressed as M_t_/M∞ = Kt^n^, where M_t_/M∞ represents the fraction of drug released at time t, K is the release rate constant, and *n* is the release exponent [31,32,33]. The in vitro drug release study was performed using a single microneedle patch at 37 °C.

#### 2.3.14. Ex Vivo Drug Release

Porcine skin was utilized to determine the ex vivo drug release of benzhydroxamic acid using a Franz diffusion cell. The excised pig skin was placed in standardized phosphate-buffered saline (PBS, pH 7.4) at 32 °C for 30 min to reflect the normal skin conditions. Further, the skin was mounted in the diffusion cell with the outer layer facing the donor compartment, comprising the MN formulation. The receptor compartment consisted of 5 mL of PBS continuously stirred at 600 rpm, optimized at 32 °C along a 1.77 cm^2^ area present for diffusion [23,24]. The equivalent quantity of fresh PBS was replaced each time on withdrawal of the sample. At predetermined time intervals, the samples were evaluated to determine the concentration of benzhydroxamic acid permeated through skin using a UV-visible spectrophotometer. The ex vivo permeation study was performed using a single microneedle patch at 32 °C.

#### 2.3.15. Statistical Analysis

Quantitative data obtained from experiments performed using three independent samples (*n* = 3) were expressed as mean ± standard deviation (SD), where applicable.

## 3. Results and Discussion

### 3.1. Design and Fabrication of 3D-Printed Master Mold and Reverse PDMS Microneedle Molds

The 3D-printed master microneedle mold was successfully fabricated with well-defined geometric features and was subsequently replicated using PDMS to produce a negative micromold with uniform and sharply defined cavities. The selected geometry provided a practical balance between needle sharpness and structural robustness. The small apex radius was intended to facilitate insertion, while the larger base radius provided structural support. The 450 µm inter-needle spacing provided adequate separation between adjacent needles and facilitated uniform cavity replication. The successful reproduction of the designed geometry in the PDMS micromold and the formation of intact microneedles supported the suitability of the selected design for the present formulation.

### 3.2. Rheological Behavior

The rheological characteristics of PVA, PVP, and benzhydroxamic acid-loaded PVA/PVP polymeric solutions were examined for their compatibility in the development of MN fabrication. They were evaluated at a shear range of 0.1–100 s^−1^. It was noticed that, on increasing the shear rate, there was a decrease in the viscosity observed in all the solutions; hence, they were recorded to have non-Newtonian pseudoplastic behavior. It was determined that shear-thinning behavior was best suited for microneedle manufacturing, and viscosity regulated the optimized filling of the mold while maintaining structural stability.

The highest viscosity was generated through PVA solution at all ranges of shear rate. The viscosity decreased from 5.82 Pa·s at 0.1 s^−1^ to 2.18 Pa·s at 100 s^−1^. The PVA solution contained extensive intermolecular hydrogen bonding and polymer chain entanglement developed from the high concentration of hydroxyl-rich PVA chains associated to produce high viscosity, whereas the PVP solution was estimated to produce lower viscosity in comparison to the PVA solution. The PVP solution displayed viscosity values ranging from 3.45 Pa·s to 1.44 Pa·s, possibly due to the deformable polymeric structure and reduced intermolecular interactions in the PVP solution.

**Figure 3:**
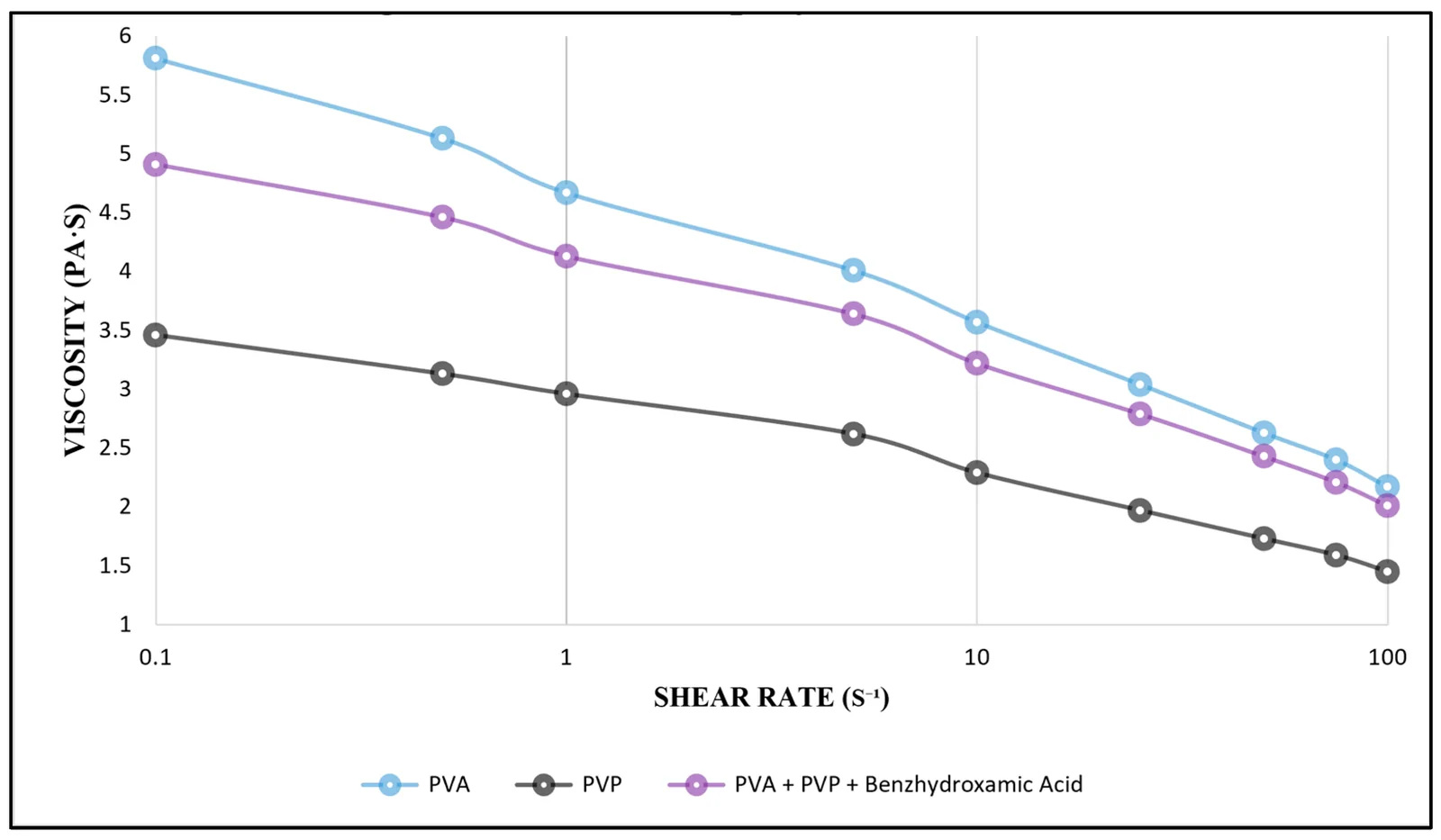
The graphical representation of the rheological behavior of the polymeric solution displays viscosity values of the benzhydroxamic acid-loaded PVA/PVP formulation (0.1–100 s^−1^).

The intermediate viscosity values were found in the benzhydroxamic acid-loaded PVA/PVP formulation. The values extended from 4.91 Pa·s at 0.1 s^−1^ to 2.01 Pa·s at 100 s^−1^, as denoted in Figure 3. This indicated effective incorporation of benzhydroxamic acid in the polymeric matrix without compromising manufacturing efficiency. The observed viscosity profile may be attributed to intermolecular interactions between benzhydroxamic acid and the PVA/PVP polymeric matrix, which may contribute to the observed flow behavior. Hence, it maintained optimized flow properties for the fabrication of microneedles.

Similar shear-thinning behavior has been reported for PVA/PVP-based dissolving microneedle formulations, where the decrease in viscosity with increasing shear rate was considered favorable for micromold filling and reproducible microneedle formation [15]. In comparison, the present benzhydroxamic acid-loaded PVA/PVP formulation exhibited an intermediate viscosity range of 4.91–2.01 Pa·s over 0.1–100 s^−1^, indicating suitable flow behavior for micro-molding filling.

### 3.3. Morphological Properties

**Figure 4:**
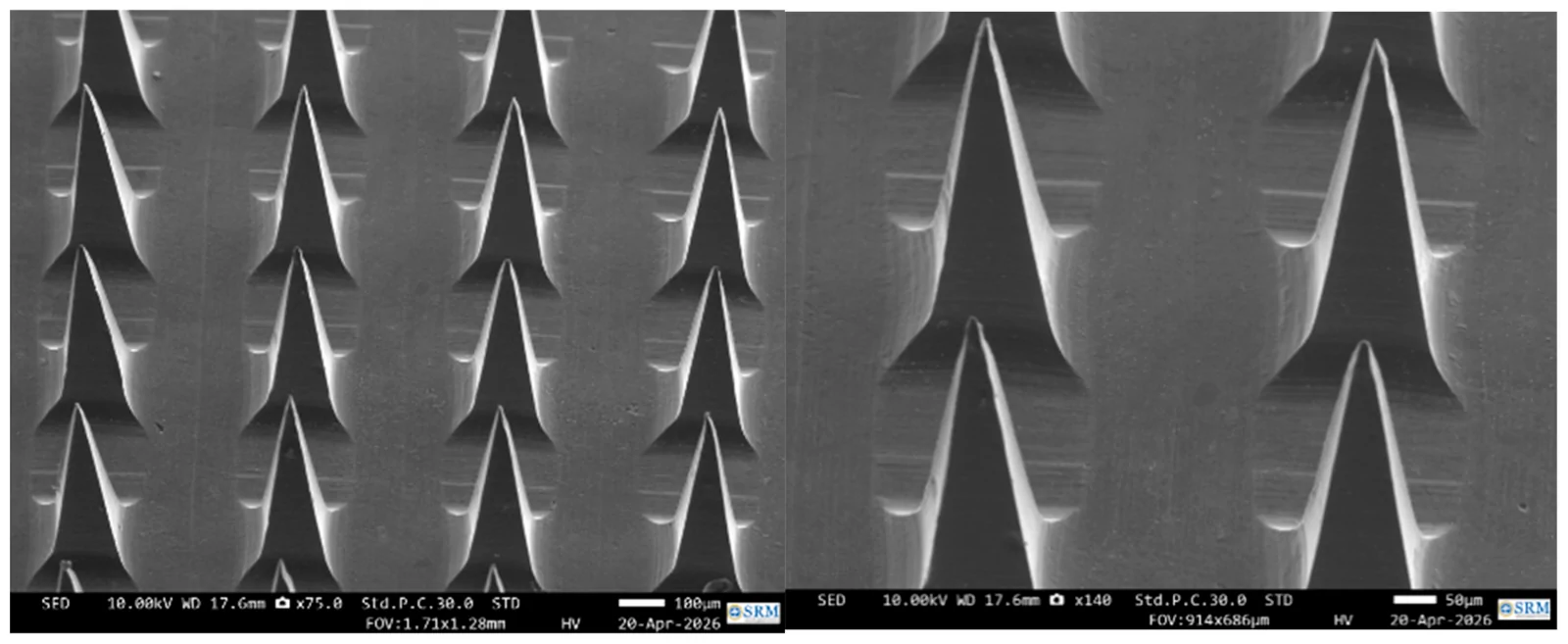
FESEM images showing the morphology and structural integrity of the benzhydroxamic acid-loaded dissolving microneedle array.

The dimensional stability and uniformity framework of the formulated dissolving microneedles were analyzed using scanning electron microscopy. Figure 4 shows the pyramidal architecture of the microneedle, with sharp tips and smooth surfaces, which was revealed to produce reliable reproducibility of model geometry during micro-molding fabrication. The micrographs determined that the fabricated microneedle patch consisted of well-organized microneedle arrays along a systematic needle distribution having geometrical dimensions throughout the patch. There were no observable defects, such as cracks, tip breakage, bending, collapse, cracking, or surface regularities. Hence, this indicated a favorable choice of polymer in the formulation of microneedles. The needle withstood the geometrical integrity and stability upon casting in the drying process, supporting the mechanical strength of the dissolving matrix. The elimination of manufacturing-related imperfections strengthens effective mold filling and uniform polymer distribution within the cavities of the micro-needle mold. Comparable, well-defined PVA/PVP microneedle structures have been reported previously [15,19]. These findings indicate that the selected PVA/PVP matrix and micromolding process were suitable for producing geometrically intact microneedle arrays.

### 3.4. Uniformity of Thickness, Width, and Weight Variation

Geometrical uniformity of the benzhydroxamic acid-loaded dissolving microneedle is estimated by measuring thickness, width, and weight variation. The established microneedle displayed a thickness of 0.51 ± 0.02 mm, a width of 10.21 ± 0.18 mm, and a weight of 17.9 ± 2.4 mg, as presented in Table 1. These results indicated consistent dimensional and mass characteristics among the evaluated patches, supporting the reproducibility of the fabrication process. These dimensional values were comparable to those reported for other PVA/PVP dissolving microneedle patches. For example, a recently reported 3D-printing-assisted PVA/PVP microneedle system exhibited a patch thickness of 0.50 ± 0.005 mm, width of 10.00 ± 0.01 mm, and weight of 18.1 ± 0.1 mg [15]. The close agreement in dimensional characteristics suggests that the present 3D-printing-assisted mold fabrication approach provides comparable dimensional reproducibility.

### 3.5. Folding Endurance

The folding endurance test displayed the flexibility and mechanical stability of the backing layer of microneedles. The test displayed 221 ± 6-fold, which indicated ideal flexibility and resistance to repeated mechanical stress of benzhydroxamic acid-loaded dissolving microneedles. There were no visible cracks, fractures, or structural degradation observed on the backing layer. The increase in the folding endurance might be due to the film-forming and elastic properties of the PVA/PVP polymer matrix. These results indicate satisfactory flexibility for processing, maintenance, and application while preserving structural integrity. The folding endurance observed in the present study was slightly higher than that reported for a recently developed PVA/PVP-based dissolving microneedle system, which exhibited a folding endurance of 214 ± 4 folds [15]. This comparable performance indicates that the PVA/PVP matrix provides adequate flexibility and resistance to mechanical deformation during handling and application.

### 3.6. Swelling Study

The swelling behavior was assessed to understand the hydration property of the benzhydroxamic acid-loaded dissolving microneedle. The study was performed in phosphate-buffered saline (pH 5.5). The swelling index was established to be 46.4 ± 0.5%, which revealed considerable water uptake by PVA/PVP polymers. The observed swelling indicates substantial hydration of the PVA/PVP matrix, which may facilitate subsequent polymer dissolution and drug release following exposure to an aqueous environment.

### 3.7. Mechanical Strength

The axial compression test evaluated the structural robustness of the needle under insertion force without causing fracture or any structural deformity. The microneedles exhibited a compression force of 3.5 ± 0.01 N/needle, indicating adequate resistance to axial deformation under the applied compression conditions. The compression force of 3.5 ± 0.01 N/needle was comparable to that reported for a 3D-printing-assisted PVA/PVP dissolving microneedle system, which exhibited a compression force of approximately 3.3 ± 0.15 N/needle [15]. The comparable compression-force values indicate that the present microneedle arrays possessed sufficient mechanical strength to maintain structural integrity during handling and insertion testing.

### 3.8. Tensile Strength

The tensile strength of the microneedle patches was 3.84 ± 0.21 MPa, indicating adequate mechanical stability and flexibility of the polymeric matrix. The tensile strength observed in the present study was comparable to previously reported PVA/PVP-based dissolving microneedle systems. A recent study using a PVA/PVP matrix reported a tensile strength of 3.21 ± 0.03 MPa, while another PVA/PVP microneedle system reported an ultimate tensile strength of approximately 4.3 ± 0.77 MPa [15,33]. Thus, the present formulation falls within the range reported for PVA/PVP-based microneedle systems, indicating adequate mechanical integrity for handling and application.

### 3.9. Insertion Study

**Figure 5:**
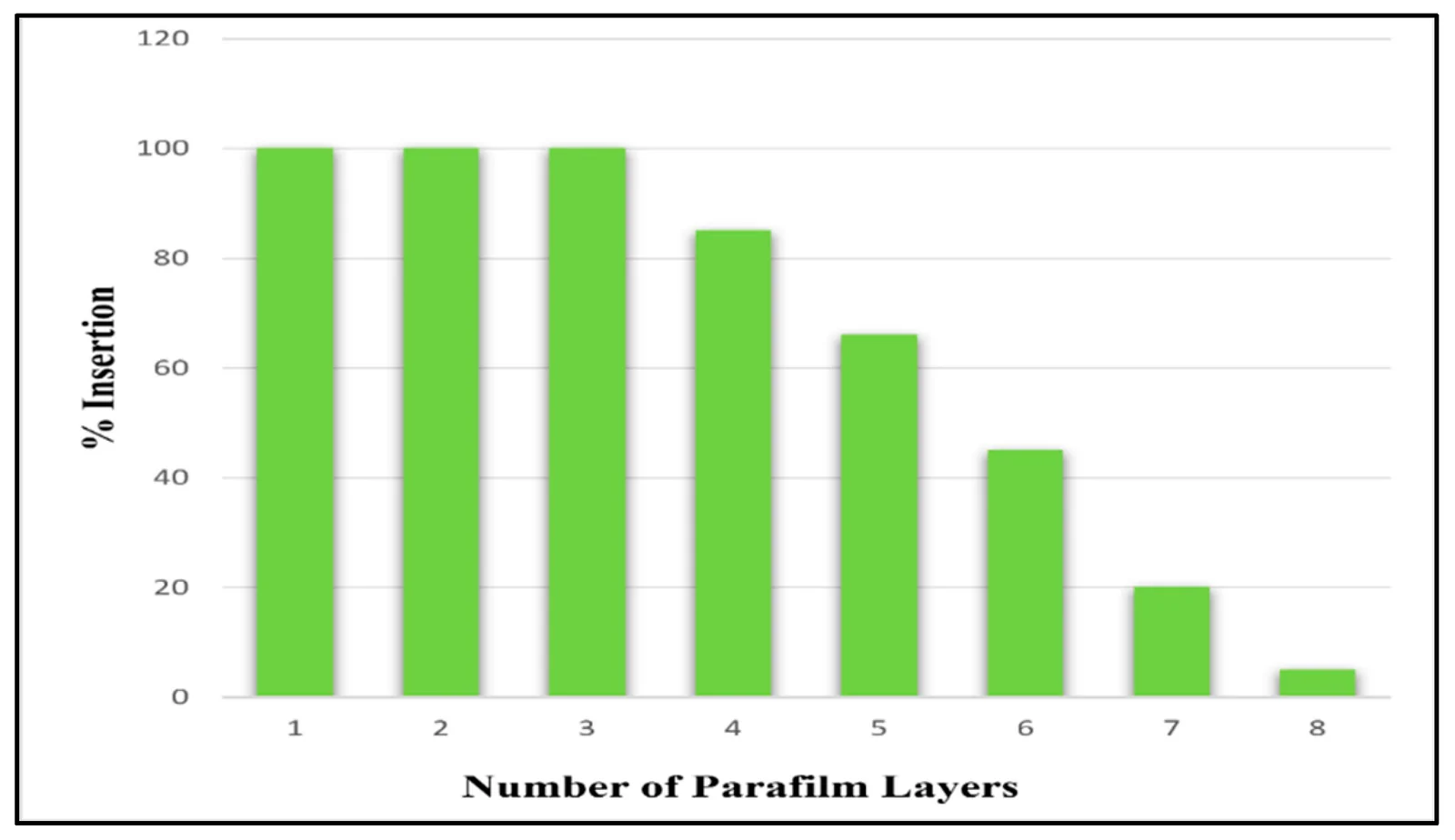
Graphical presentation of insertional capability of benzhydroxamic acid-loaded dissolving microneedles evaluated using the Parafilm^®^ M skin-simulant model.

The insertional studies demonstrated the penetration capability and mechanical integrity of the benzhydroxamic acid-loaded dissolving microneedles using a multilayer Parafilm^®^ model. As represented in Figure 5, the formulated microneedles penetrated the first 3 layers with complete 100% penetration efficiency. The penetration rate decreased upon increasing the number of layers. The penetration rate obtained at the fourth, fifth, sixth, seventh, and eighth layers was 88%, 70%, 48%, 24%, and 8%, respectively.

The decrease in penetration percentage with increasing Parafilm^®^ M layer number indicates progressively greater resistance to penetration as the effective barrier thickness increased. However, the penetration rate of the needles into the multiple layers indicated the sufficient sharpness and structural robustness of the fabricated arrays.

Previous studies have reported that penetration through approximately three to four Parafilm^®^ M layers can serve as an indicator of sufficient microneedle insertion capability [15]. In the present study, the benzhydroxamic acid-loaded microneedles completely penetrated the first three layers and partially penetrated the fourth layer, demonstrating insertion performance comparable to previously reported dissolving microneedle systems. Therefore, these results indicate that the benzhydroxamic acid-loaded microneedle formulation exhibited satisfactory insertion capability and maintained structural integrity during insertion.

### 3.10. Analytical Method for Benzhydroxamic Acid Quantification

**Figure 6:**
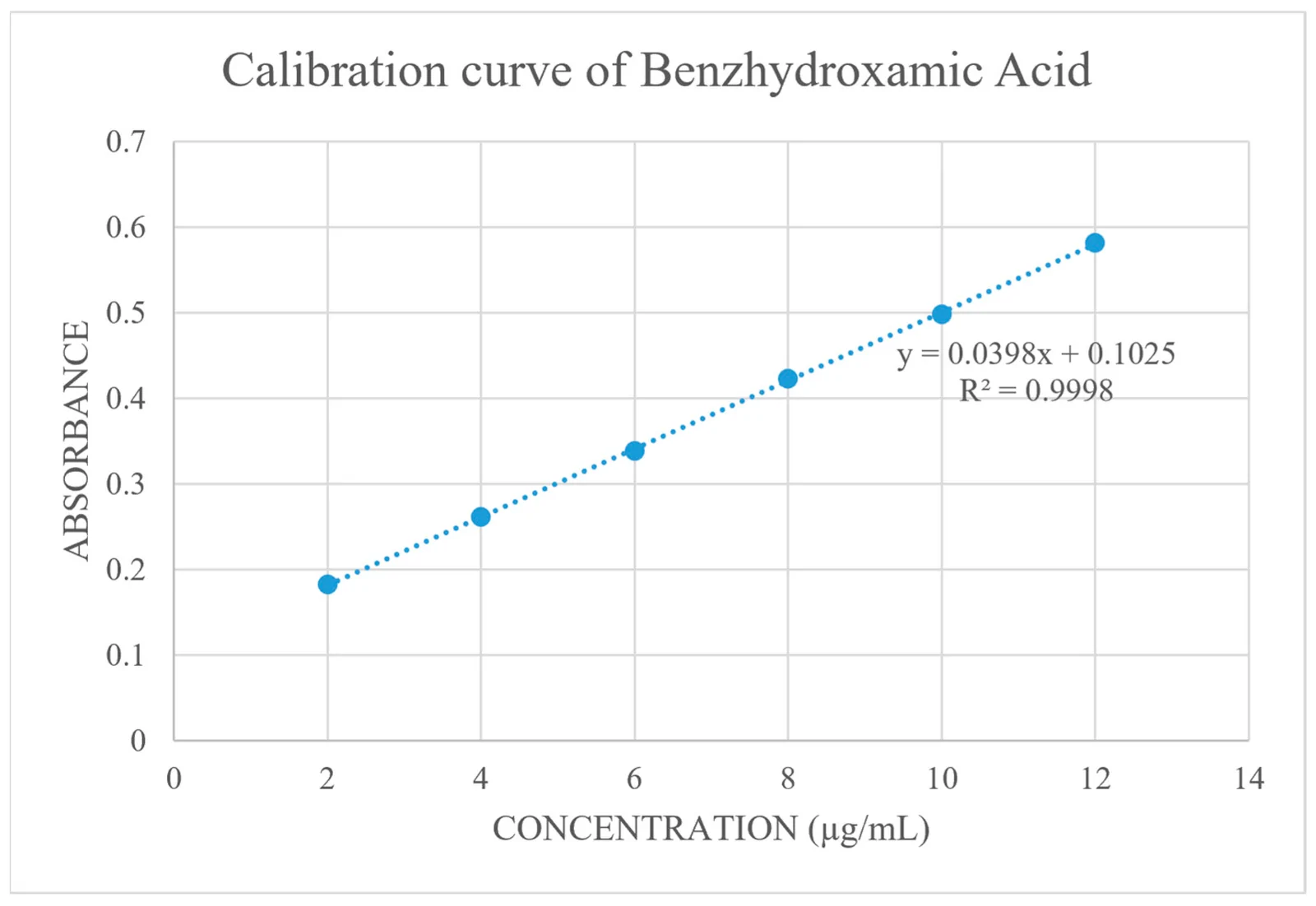
Calibration curve of benzhydroxamic acid showing the linear relationship between concentration (2–12 µg/mL) and absorbance measured at 230 nm.

An over 2–12 µg/mL proportional relationship between concentration and absorbance was displayed in the calibration curve of the benzhydroxamic acid. Figure 6 represents the regression equation, y = 0.0398x + 0.1025, with a correlation coefficient (R^2^) of 0.9998. This indicated excellent linearity throughout the selected concentration range. The correlation coefficient confirmed the reliability and suitability of the developed UV spectrophotometric method in the quantification of benzhydroxamic acid. The established calibration curve was subsequently used for quantification of benzhydroxamic acid during drug-loading and in vitro drug-release studies.

### 3.11. Drug Loading Efficiency

The BHA-loaded dissolving microneedles exhibited a drug-loading efficiency of 94.6 ± 0.35%, indicating the efficient incorporation of the drug into the PVA/PVP matrix. The high loading efficiency may be attributed to the compatibility of BHA with the hydrophilic polymeric matrix and the controlled casting process, which facilitated retention of the drug within the microneedle cavities during fabrication. The observed loading efficiency supports the reproducibility of drug incorporation within the developed microneedle formulation.

### 3.12. FTIR

**Figure 7:**
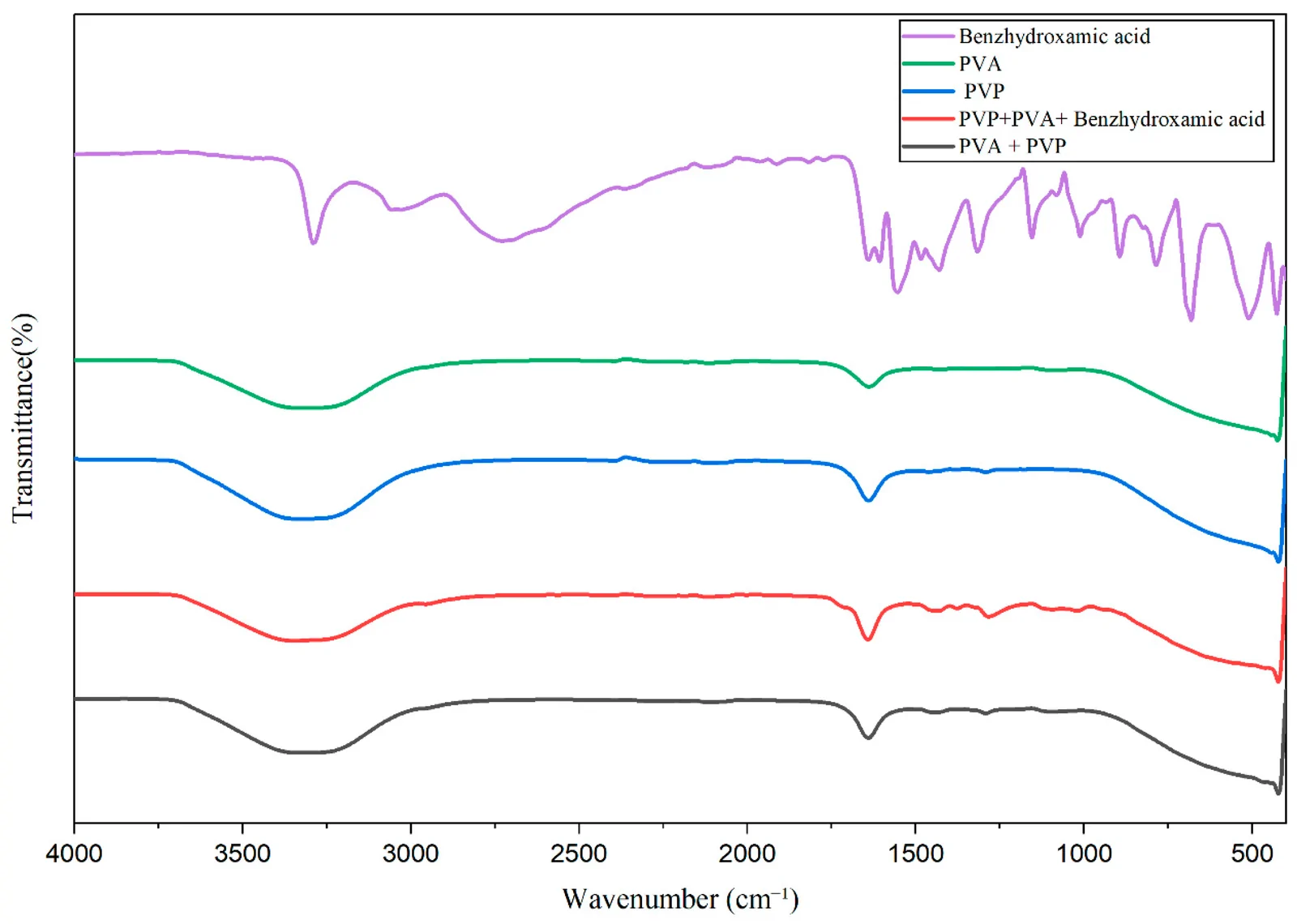
FTIR spectrum of drug, polymers, and microneedle formulation.

FTIR was mainly performed to analyze the suitability between the benzhydroxamic acid and polymeric compounds such as PVA and PVP, which are used to fabricate dissolving microneedles. Figure 7 displays the FTIR spectrum of pure benzhydroxamic acid, individual polymers, polymeric matrix, and benzhydroxamic acid-loaded dissolving microneedles. The pure benzhydroxamic acid spectrum displayed distinctive absorption bands associated with functional groups encompassing a broad peak in the region of 3200 to 3400 cm^−1^ linked to O-H and N-H stretching vibrations along with bands associated with C=O stretching and C-N functional groups. The PVA spectrum provided a broad absorption band around 3300 to 3400 cm^−1^ connected to O-H stretching vibration and defined peaks linked to C-H and C-O stretching. Likewise, the PVP spectrum also generated a distinctive absorption band around 1650 cm^−1^ related to the C=O stretching vibration of the pyrrolidine ring. The drug-loaded dissolving microneedle exhibited a similar spectrum with defined peaks of both benzhydroxamic acid and polymeric matrix, with minor shifts in the peak positions and intensities. These minor differences may be attributed to possible intermolecular interactions between benzhydroxamic acid and the PVA/PVP polymeric matrix. It was observed that there was neither the presence of any new absorption peak nor the absence of characteristic peaks in the formulation peak. Hence, this indicated the compatibility and stability of benzhydroxamic acid during the process of microneedle fabrication. These findings support the compatibility of benzhydroxamic acid with the PVA/PVP matrix, with no major spectral evidence of chemical incompatibility. The observed preservation of the characteristic functional groups is consistent with previous reports of PVA/PVP-based dissolving microneedles, in which FTIR analysis demonstrated compatibility between the incorporated drug and the polymeric matrix without the appearance of new characteristic peaks [15]. This supports the suitability of the PVA/PVP matrix for incorporating benzhydroxamic acid without major chemical incompatibility.

### 3.13. X-Ray Diffraction (XRD) Analysis

**Figure 8:**
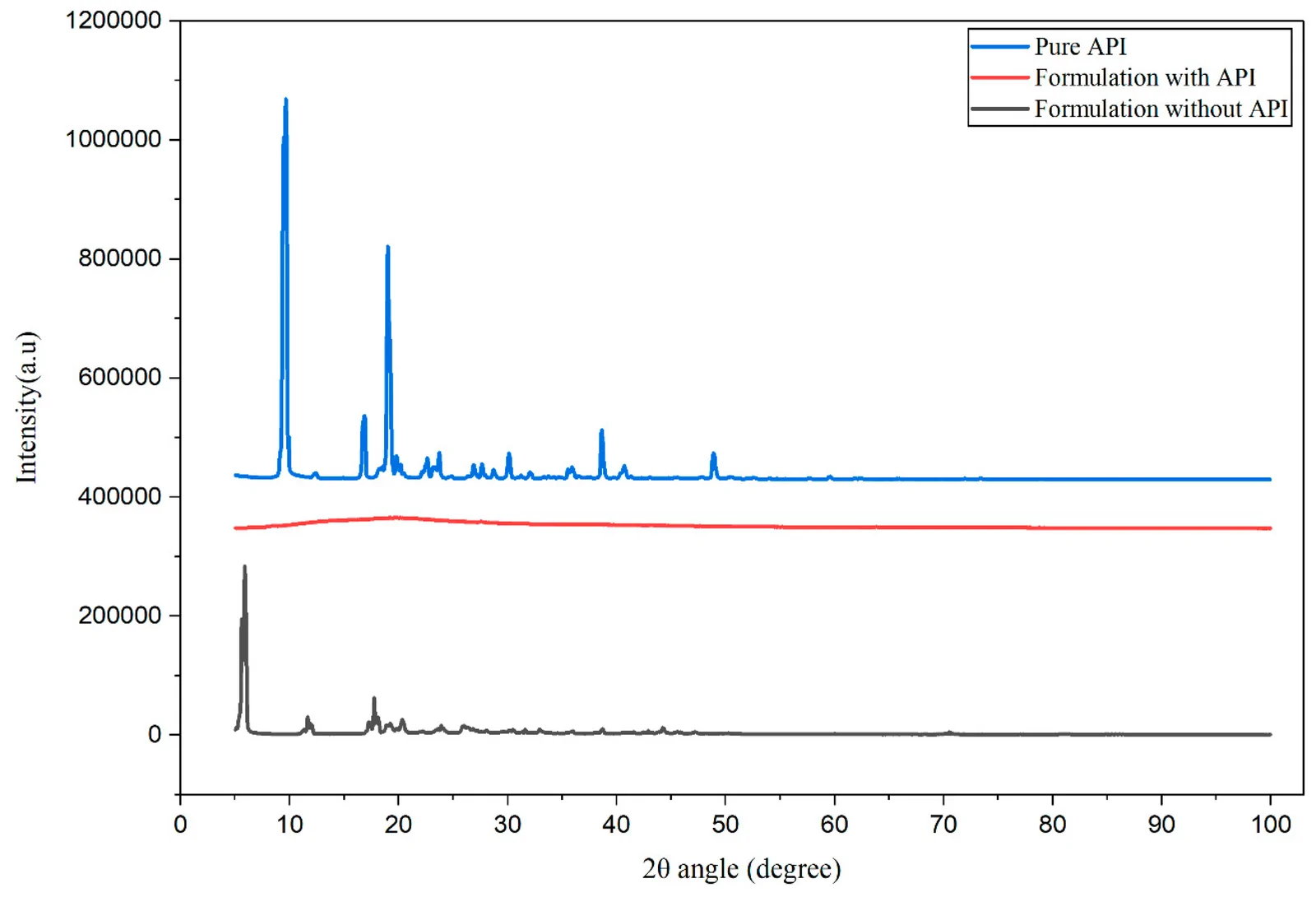
X-ray diffraction (XRD) patterns of pure benzhydroxamic acid, the blank PVA/PVP formulation, and the benzhydroxamic acid-loaded dissolving microneedle formulation.

XRD analysis was conducted to assess the crystalline characteristic of benzhydroxamic acid and to examine the changes in its morphological form on incorporation into a PVA/PVP matrix. Figure 8 displays the diffraction patterns of pure benzhydroxamic acid, the blank formulation (without API), and the benzhydroxamic acid-loaded formulation. The result showed the pure benzhydroxamic acid developed several intense and sharp diffraction peaks at specific 2θ values, reflecting its high crystalline nature, whereas the PVA/PVP formulations presented hollow peaks with decreased intensity, indicating the characteristic of an amorphous polymeric matrix. The benzhydroxamic acid-loaded formulation exhibited strong peaks of the pure drug following the broadening of peaks and the appearance of a predominantly diffuse diffraction profile.

The reduction in the strong peak intensity of the drug-loaded formulation denotes a decrease in the crystalline form of benzhydroxamic acid within the polymeric matrix. This might be due to the interaction between the drug and the PVA/PVP network. Hence, the increase in the amorphous nature would enhance the solubility and dissolution behavior. Moreover, there was no presence of any new peaks, absence of any undesirable crystalline impurities, or incompatibility between the drug and excipients. The XRD findings support the incorporation of benzhydroxamic acid into the PVA/PVP matrix and suggest a reduction in its apparent crystallinity following formulation. Similar reductions in drug crystallinity following incorporation into hydrophilic polymeric microneedle matrices have been reported previously. Such changes are generally associated with molecular dispersion of the drug within the polymer network and may contribute to improved dissolution behavior [15]. The present XRD findings therefore support the successful incorporation and dispersion of benzhydroxamic acid within the PVA/PVP matrix.

### 3.14. In Vitro Drug Release Study

The in vitro release of the benzhydroxamic acid-loaded formulation was investigated for 24 h. A biphasic pattern of release kinetics was observed in the developed formulation, primarily the drug burst, followed by sustained release over a period.

**Figure 9:**
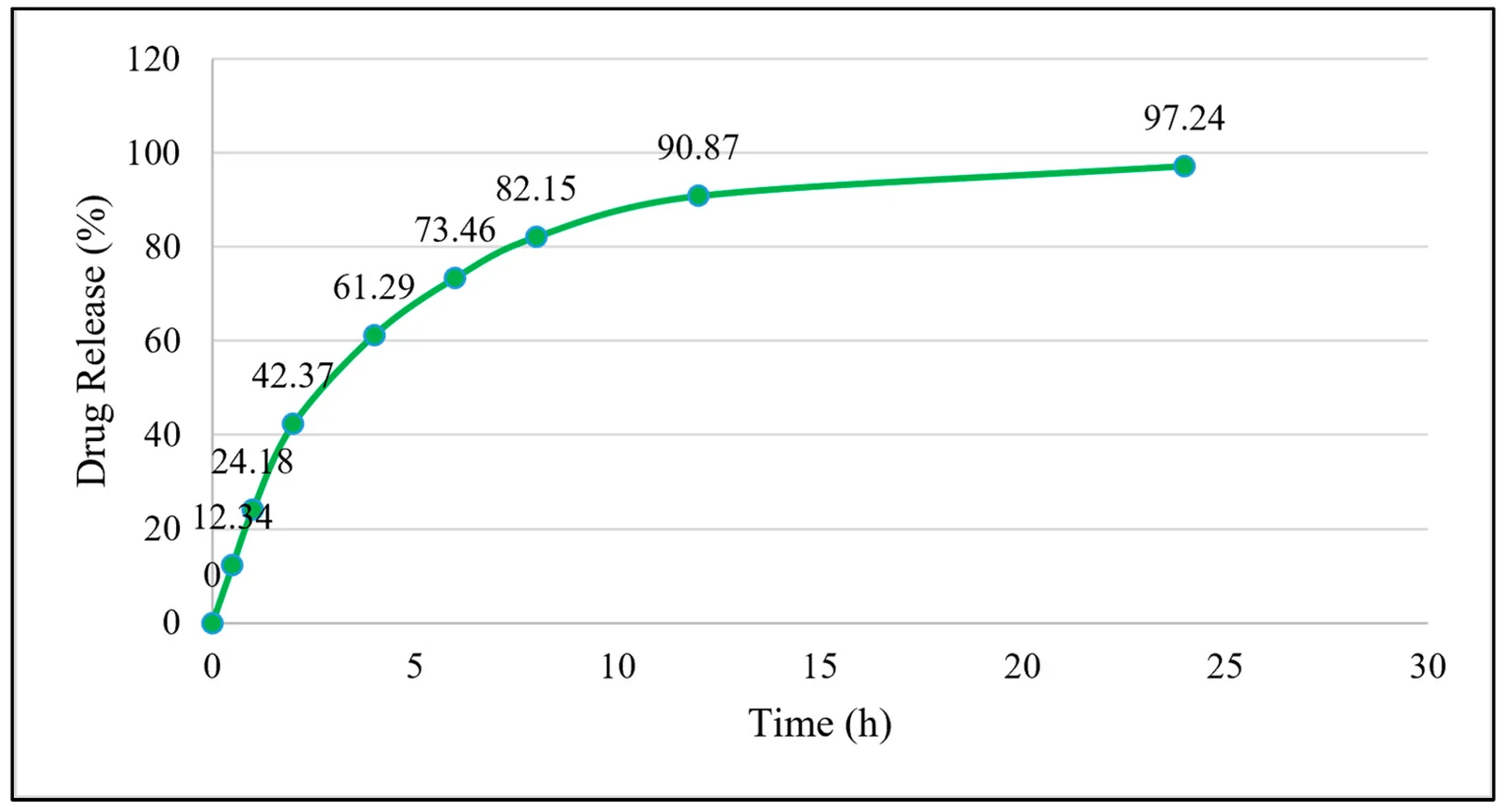
In vitro drug release profile of benzhydroxamic acid from the PVA/PVP dissolving microneedle formulation over 24 h.

Figure 9 shows that at the initial 0.5 h, 12.34% release was observed; subsequently, there was an increase in the release of 24.18% at 1 h and 42.37% at the 2 h period. Thereafter, a cumulative drug release was observed at 4 h, with 61.29% release, suggesting enhanced hydration and dissolution of the hydrophilic PVA/PVP matrix. The later sustained release phase of drug release was indicated as having 73.46% at 6 h, 82.15% at 8 h, and 90.87% at 12 h. At the end of the 24 h period, 97.24% of the drug release was observed, showcasing the complete drug release from the microneedle matrix.

The initial burst of the drug is initiated through the needle tip and upon the surface, whereas the sustained release patterns were regulated by diffusion of benzhydroxamic acid through the hydrated polymer network and continuous dissolution of the polymeric matrix.

The drug-release profile was further evaluated using zero-order, first-order, Higuchi, and Korsmeyer–Peppas kinetic models (Table 2). The Korsmeyer–Peppas model showed the highest coefficient of determination (R^2^ = 0.9973), followed by the first-order model (R^2^ = 0.9650), the Higuchi model (R^2^ = 0.8688), and the zero-order model (R^2^ = 0.6702). The release exponent (*n*) obtained from the Korsmeyer–Peppas model was 0.890, suggesting anomalous/non-Fickian release behavior, indicating that both diffusion and polymer-related processes may contribute to the observed drug-release profile.

These findings indicate that the developed formulation exhibited a sustained drug-release profile and support its feasibility for further investigation as a transdermal delivery platform.

### 3.15. Ex Vivo Drug Release

**Figure 10:**
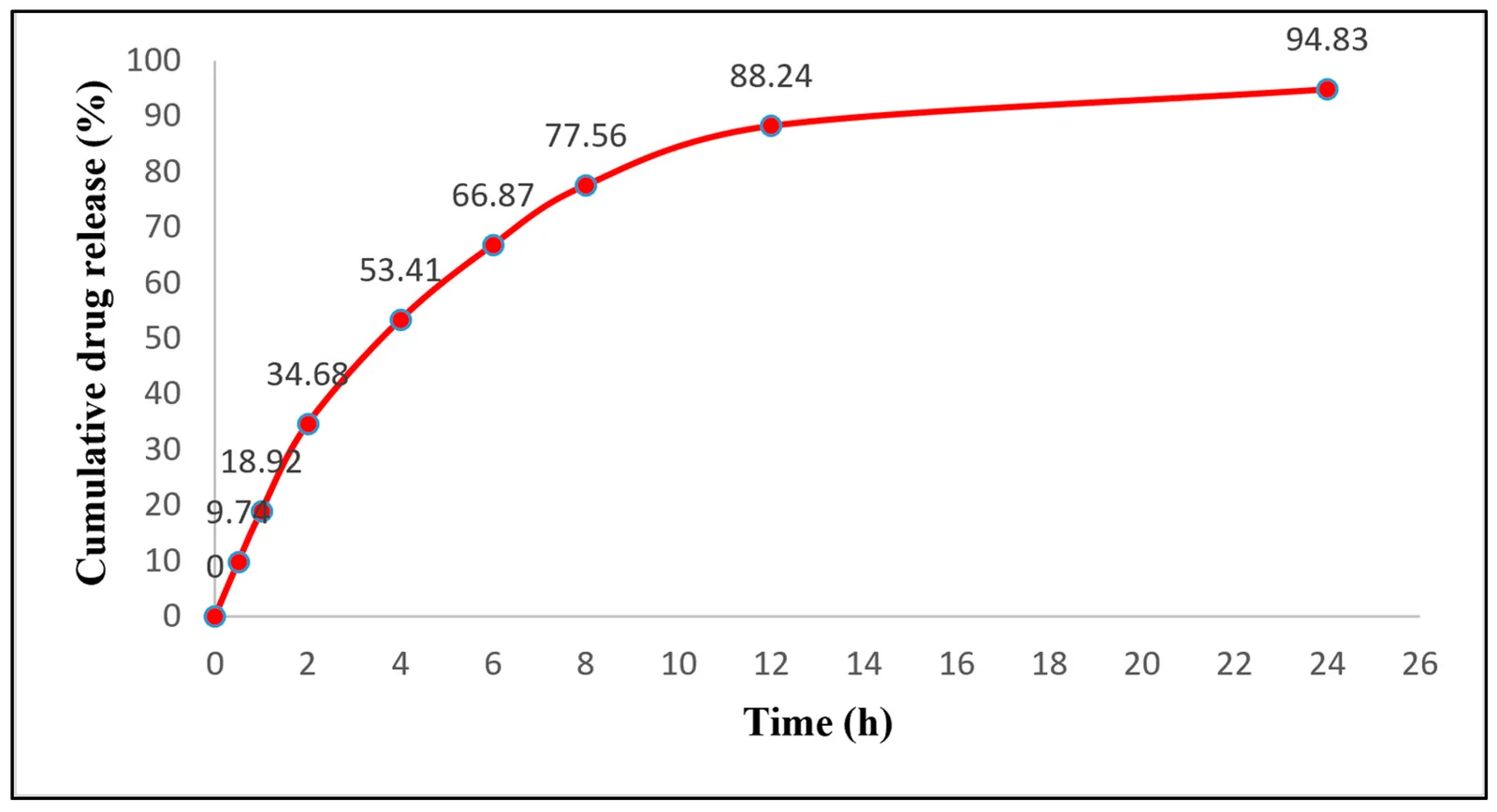
Ex vivo drug release profile of benzhydroxamic acid from the PVA/PVP dissolving microneedle formulation over 24 h.

The ex vivo drug release of the fabricated formulation was performed using excised skin on a Franz diffusion cell apparatus. Figure 10 displays the cumulative percentage of permeation in the skin over a 24 h period.

The formulation exhibited sustained release of the drug with initial rapid permeation of 9.70% and 18.92% drug permeation at 0.5 h and 1 h, respectively. Further, the permeation of the drug increased to 34.68% at 2 h and 53.41% at 4 h, followed by 66.87% at 6 h, 77.56% at 8 h, 88.24% at 12 h, and 94.83% at 24 h.

The increased permeation of the drug in the initial hours exhibited transient microchannels in the skin due to the formulated dissolving microneedles, which effectively bypassed the stratum corneum and facilitated rapid drug transport, whereas the sustained release was observed due to dissolution of the PVA/PVP polymer matrix and continuous diffusion of benzhydroxamic acid through the hydrated skin layers.

The enhanced permeation observed with the microneedle formulation indicates that transient microchannels generated during insertion facilitated transport of benzhydroxamic acid across the skin barrier. These findings support the feasibility of the developed PVA/PVP dissolving microneedles for transdermal delivery of benzhydroxamic acid.

### 3.16. Study Limitations and Future Perspectives

The present study represents a formulation-development and proof-of-concept investigation and has certain limitations. Although the developed microneedles demonstrated satisfactory physicochemical and mechanical characteristics, effective insertion into the Parafilm^®^ M skin-simulant model, high drug-loading efficiency, in vitro drug release, and ex vivo skin permeation, therapeutic efficacy was not evaluated in a disease-specific biological model. In addition, pharmacokinetic and pharmacodynamic studies were not performed to establish drug exposure and therapeutic effects following microneedle administration. Therefore, the current findings demonstrate the feasibility of the microneedle platform for BHA delivery rather than establishing therapeutic efficacy. Future studies should include biological skin insertion and dissolution studies, followed by pharmacokinetic, pharmacodynamic, safety, and efficacy evaluation in appropriate animal models.

## 4. Conclusions

This study successfully developed a benzhydroxamic acid-loaded PVA/PVP dissolving microneedle system using a 3D-printing-assisted micromolding approach. The developed microneedles exhibited uniform morphology, satisfactory mechanical properties, efficient drug loading, and effective insertion into the Parafilm^®^ M skin-simulant model. FTIR and XRD analyses supported the compatibility and incorporation of benzhydroxamic acid within the polymeric matrix. The formulation also demonstrated sustained in vitro drug release and ex vivo skin permeation over 24 h. Collectively, these findings establish the feasibility of the developed platform for transdermal delivery of benzhydroxamic acid and provide the foundation for subsequent pharmacokinetic, pharmacodynamic, safety, and therapeutic efficacy studies.

## Figures and Tables

**Table 1:** Morphological and functional assessment of benzhydroxamic acid-loaded dissolving microneedles. Data are presented as mean ± SD.

Parameter	Results
Thickness (mm)	0.51 ± 0.02
Width (mm)	10.21 ± 0.18
Weight (mg)	17.9 ± 2.4
Folding endurance (no. of folds)	221 ± 6
Swelling index (%)	46.4 ± 0.5
Compression force (N/needle)	3.5 ± 0.01
Tensile strength (MPa)	3.84 ± 0.21

**Table 2:** Release kinetic analysis of BHA-loaded dissolving microneedles.

Kinetic Model	R^2^
Zero-order	0.6702
First-order	0.9650
Higuchi	0.8688
Korsmeyer–Peppas	0.9973

## Data Availability

The data presented in this study are available within the article.
